# The clinical outcomes of COVID‐19 in HIV‐positive patients: A systematic review of current evidence

**DOI:** 10.1002/iid3.497

**Published:** 2021-07-29

**Authors:** SeyedAhmad SeyedAlinaghi, Amirali Karimi, Mehrzad MohsseniPour, Alireza Barzegary, Seyed Peyman Mirghaderi, Amirata Fakhfouri, Solmaz Saeidi, Armin Razi, Hengameh Mojdeganlou, Marcarious M. Tantuoyir, Amir Masoud Afsahi, Esmaeil Mehraeen, Omid Dadras

**Affiliations:** ^1^ Iranian Research Center for HIV/AIDS, Iranian Institute for Reduction of High Risk Behaviors Tehran University of Medical Sciences Tehran Iran; ^2^ School of Medicine Tehran University of Medical Sciences Tehran Iran; ^3^ School of Medicine Islamic Azad University Tehran Iran; ^4^ Department of Nursing University of Medical Sciences Khalkhal Iran; ^5^ Internal Medicine Department Tehran University of Medical Sciences Tehran Iran; ^6^ Department of Pathology Urmia University of Medical Sciences Urmia Iran; ^7^ Biomedical Engineering Unit University of Ghana Medical Center (UGMC) Accra Ghana; ^8^ Department of Radiology University of California, San Diego (UCSD) San Diego California USA; ^9^ Department of Health Information Technology Khalkhal University of Medical Sciences Khalkhal Iran; ^10^ AMAD Research Institute Supreme National Defense University Tehran Iran; ^11^ Department of Global Health and Socioepidemiology, Graduate School of Medicine Kyoto University Kyoto Japan

**Keywords:** clinical outcome, COVID‐19, HIV/AIDS, SARS‐CoV‐2, severity

## Abstract

**Introduction:**

Patients with chronic underlying diseases are more susceptible to coronavirus disease 2019 (COVID‐19) complications. Recent studies showed people living with HIV (PLWH) are not at greater risk than the general population. Few studies have reviewed the impacts of COVID‐19 on PLWH. The purpose of this systematic review was to investigate the impact of COVID‐19 on patients infected with HIV.

**Methods:**

We executed a systematic search using four databases of PubMed, Scopus, Science Direct, and Web of Science and screened the records in two steps based on their title/abstract and full text. This study follows the Preferred Reporting Items for Systematic Reviews and Meta‐Analyses (PRISMA) checklist to elevate the validity and reliability of its results.

**Results:**

We reviewed 36 studies. The patients' age was above 20 years in all studies. In almost all studies, the inflammatory parameters were reported high. In most of the studies, all HIV patients completely recovered from the COVID 19 infection. Although CD4 count was not recorded in all studies, the minimum level was reported as 12 cells/µl.

**Conclusion:**

Based on the current review, we concluded that HIV patients at advanced stages (3 or 4) of the disease, whose CD4 counts are low, may show less severe COVID‐19 infection symptoms. Similarly, Interference can reduce the severity of immune reactions and subsequent cytokine storms and consequently mitigate the symptoms. Therefore, in most of the studies, the majority of HIV patients showed no severe symptoms and completely recovered from COVID 19 infection.

## 1 INTRODUCTION

At the end of December 2019, cases of a highly contagious infectious disease had been reported in Wuhan, China.^1^, ^2^, ^3^, ^4^, ^5^, ^6^, ^7^ Severe acute respiratory syndrome coronavirus 2 (SARS‐CoV‐2) is a new strain of coronavirus and belongs to the Beta coronavirus genus of the Coronaviradae family. Due to the high interpersonal transmission rate, soon SARS‐CoV‐2 spread globally and turned into a global pandemic.^6^, ^8^, ^9^, ^10^ The World Health Organization (WHO) announced its concern about the novel coronavirus on January 30, 2020, and declared the highest level of alarm as a public health emergency.^11^ The COVID‐19 outbreak was been declared as a global pandemic by WHO on March 11, 2020.^12^ As of May 30th, a total of 169 million cases of infected patients were reported around the world and the number of deaths reached 3.52 million.^13^ Coronavirus disease 2019 (COVID‐19) can affect different organs in the human body. The most common complications of SARS‐CoV‐2 are respiratory failure and Acute Respiratory Distress Syndrome (ARDS).^14^ Risk factors of COVID‐19 are related to the type of immune system response and host factors such as age, gender, underlying diseases, and so on.^15^, ^16^, ^17^, ^18^

Patients with chronic underlying diseases can experience COVID‐19 complications more than the general population, although recent studies showed people living with HIV (PLWH) are not at greater risk than the general population.^19^, ^20^ HIV attacks a specific type of immune system cells known as a CD4 helper cell and destroys them. When the CD4 count drops below 200 cells/µl, the patient will have progressed to AIDS. According to the Joint United Nations Programme on HIV/AIDS (UNAIDS), the number of PLWH around the world was 37.9 million,^21^ and unfortunately, around 12 million of them are not receiving antiretroviral therapy (ART). A recent study revealed 86% of HIV patients receiving ART, have a positive response to it and this leads to undetectable viral load. In conclusion, patients who respond to ART and maintain treatment are not immunocompromised.^19^

Few studies have reviewed the impacts of COVID‐19 on PLWH. There is yet no certain answer whether patients infected with HIV are at greater risk of severe illness and worse outcomes. We still need to know whether there are any differences between controlled HIV infection with undetectable viral load and a CD4 count ≥200 cells/µl and uncontrolled HIV infection or AIDS in COVID‐19 outcomes. Thus, the aim of this systematic review was to investigate the outcome of COVID‐19 among patients infected with HIV.

## 2 METHODS

### 2.1 Study design

We systematically searched four databases, including PubMed, Scopus, Science Direct, and Web of Science to retrieve the related articles based on the keywords used in our search strategy. Two researchers (S. P. M. and S. S.) screened the retrieved articles using the Covidence website (https://www.covidence.org). The screening consisted of two‐step title/abstract and full‐text screening processes. Utilizing this website eased settling of the discrepancies in the inclusion process. A third researcher (A. K.) addressed the remaining discrepancies. Two researchers (H. M. and A. R.) extracted and summarized the data of the included studies.

### 2.2 Search strategy

We included the search terms for HIV/AIDS and COVID‐19 as presented below:

A. [SARS‐CoV‐2] (Title/Abstract) OR [COVID‐19] (Title/Abstract) OR [2019‐nCoV] (Title/Abstract) OR[Novel Coronavirus] (Title/Abstract)
B. [Human Immunodeficiency Virus] (Title/Abstract) OR [HIV] (Title/Abstract) OR [Acquired Immune Deficiency Syndrome] (Title/Abstract) OR [AIDS] (Title/Abstract)
C. [A] AND [B].

### 2.3 Inclusion criteria

We included all the original articles discussing the COVID‐19 status in PLWH. The exclusion criteria are the following:

(1) Review articles, letter to the editors, or other studies without original data.
(2) Ongoing studies.
(3) Irrelevant to the aims, settings, and design of this study.
(4) Abstracts, conference abstracts, errata, or other studies lacking full‐texts.

### 2.4 Quality assessment

We utilized the National Institutes of Health (NIH) tool^22^ to evaluate the quality of the studies. A researcher (A. K.) examined all the studies to ensure the quality of evidence. If an element of the criteria was insufficiently addressed, not applicable, or not reported in a study and it could not be identified indirectly, we did not allocate a score to that element. For cohort and cross‐sectional studies, 11–14 was considered good, 6‐10 fair, and 0‐5 poor. We chose 9–12, 5–8, and 0–4 for good, fair, and poor quality in the case‐control studies, respectively. The numbers were 7–9, 4–6, and 0–3 for the case series. Case reports were checked with the same checklist as the case series.

## 3 RESULTS

Most of the studies were considered of good quality (25/37). Other studies (12/37) had a fair quality, and we did not classify any studies as poor (Table 1).

**Table 1:** Quality assessment of the included studies using NIH tool

Study	Study design	Score	Quality rating (good, fair, or poor)
Blanco et al. JL^23^	Case series	7/9	Good
Brown et al.^24^	Prospective cohort	13/14	Good
Cabello et al.^25^	Case series	8/9	Good
Calza et al.^26^	Case series	7/9	Good
Calza et al.^27^	Case series	6/9	Fair
d'Ettorre et al.^28^	Case report	8/9	Good
D'Souza et al.^29^	Cohort	10/14	Fair
Boulle et al.^30^	Retrospective cohort	12/14	Good
Del Amo et al.^31^	Retrospective cohort	11/14	Good
Etienne et al.^32^	Prospective cohort	12/14	Good
Geretti et al.^33^	Prospective cohort	13/14	Good
Gudipati et al.^34^	Case series	7/9	Good
Guo et al.^35^	Retrospective cohort	10/14	Fair
Härter et al.^36^	Case series	7/9	Good
Ho et al.^37^	Case series	8/9	Good
Karmen‐Tuohy et al.^38^	Retrospective cohort	11/14	Good
Kim et al.^39^	Case report	7/9	Good
Kowalska et al.^40^	Case series	8/9	Good
Kumar et al.^41^	Case report	6/9	Fair
Menghua et al.^42^	Case report	7/9	Good
Myashita and Kuno^43^	Retrospective cohort	9/14	Fair
Mondi et al.^44^	Case series	7/9	Good
Nagarakanti et al.^45^	Retrospective cohort	10/14	Fair
Okoh et al.^46^	Case series	7/9	Good
Patel and Pella^47^	Case report	6/9	Fair
Ridgway et al.^48^	Cohort	7/14	Fair
Ruan et al.^49^	Case series	6/9	Fair
Sachdev et al.^50^	Retrospective cohort	9/14	Fair
Stoeckle et al.^51^	Retrospective cohort	11/14	Good
Swaminathan et al.^52^	Case series	7/9	Good
Tesoriero et al.^53^	Retrospective cohort	10/14	Fair
Toombs et al.^54^	Case report	8/9	Good
Vizcarra et al.^55^	Prospective cohort	11/14	Good
Wang et al.^56^	Case report	6/9	Fair
Wu et al.^57^	Case report	7/9	Good
Yang et al.^58^	Retrospective cohort	12/14	Good
Zhang et al.^59^	Case report	7/9	Good

Thirty‐six studies met the inclusion criteria. The results of these 36 studies are summarized in Tables 2 and 3. Forty percent of the studies were from the USA, 20% from China, 11. 4% from Italy, 11. 4% from Spain and the remainder were from Germany, France, UK, South Korea, Poland, and South Africa. All the articles were published between January and December 2020. The study population in the explored articles ranged from one patient (in case reports) to 3,460,932 patients in a large cohort study from South Africa (Table 2).

**Table 2:** Characteristics of studies included in the review of COVID‐19‐HIV co‐infection

ID	Study (reference)	Country	Study population	Total cases (HIV + cases)	Age (year)	Sex
1	Bhaskaran et al.^60^	UK	17,282,905 (COVID‐19 status is not mentioned)	27,480 (COVID‐19 status is NOT mentioned)	18–39 years: 6625 (24.1%) 40–49 years: 8093 (29.5%) 60–69 years: 3130 (11.4%) 70–79 years: 937 (3.4%) ≥80 years: 209 (0.8%) Median (IQR): 48 years (40–55)	Male: 17780 (64.7%) Female: 9700 (35.3%)
2	Blanco et al.^23^	Spain	5	5	40, 49, 29, 40, 31 years	Transgender: 2 (40%), Male: 3 (60%)
3	Cabello et al.^25^	Spain	63	63	Median (IQR): 46 years (37–52)	Male: 56 (88. 9%)
4	Calza et al.^26^	Italy	9	9	Median (IQR): 56.2 years (41–73)	Male: 7 (78%)
5	Calza et al.^27^	Italy	26	26	Median (IQR): 53.8 years (28–80)	Male: 19 (73%)
6	Cipolat et al.^61^	Brazil	1	1	63 years	Female
7	D'Ettorre et al.^28^	Italy	16	11	52 years	Female: 1 (100%)
8	D'souza et al.^29^	USA	3411	2078	Median (IQR): 57 years (26–94)	Male: 955(46%) female:1123 (54%)
9	Davies^62^	South Africa	3,460,932	3978	All patients were above 20 years	Male: 1682 (42.3%), female: 2296 (57.7%)
10	Del Amo et al.^31^	Spain	77,590	77590	20–39 years: 14,506 (19%) 40–49 years: 19,373 (25%) 50–59 years: 32,321 (42%) 60–69 years: 8762 (11%) 70–79 years: 2628 (3%)	Male: 58,120 (75%), female: 19,470 (25%)
11	Ettiene et al.^32^	France	54	54	Median (IQR): 54 years (47–60)	Male: 33 (61.1%), female:21 (38.9%)
12	Geretti et al.^33^	England	47,592	122	Median (IQR): HIV + group: 56 years (49–62) HIV‐ group:74 years (60–84)	Female: HIV + group: 41/121 (33. 9%) HIV‐group: 20,302/47,303 (42.9%)
13	Gudipati et al.^34^	USA	14	14	Median (IQR): 57.5 years (36–74)	Male:12 (85.7%), female:2 (14.3%)
14	Guo et al.^63^	China	1701	1701 11 confirmed COVID‐19	Mean: 42 ± 14.5 years	Male: 1484 (87.2%), female: 217 (12.8%)
15	Härter et al.^36^	Germany	33	33	Mean (*SD*): 48 years (26–82)	Male: 30 (91%), female: 3 (7%)
16	Ho et al.^37^	USA	93	93	Median (IQR): 58 years (52–65)	Male: 67 (72%), female: 23 (24. 7%) Transgender: 3 (3.2%)
17	Karmen‐Tuohy et al.^38^	USA	63	21	Not mentioned	Not mentioned
18	Kim et al.^39^	Korea	1	1	29 years	Male: 1 (100%)
19	Kowalska et al.^40^	Poland	34	34	Median: 40.5 years	Female: 10 (29.4%), Male: 24 (70.6%)
20	Kumar et al.^41^	USA	1	1	50 years	Male:1 (100%)
21	Menghua et al.^42^	China	1	1	49 years	Female: 1 (100%)
22	Miyashita et al.^43^	USA	8912	161	≤50 years: 38 51–65 years: 82 ≥66 years: 41	Male: 125(77%), female: 36 (23%)
23	Mondi et al.^44^	Italy	605	5	61, 46, 31, 55, 55 years	Male: 4 (80%), Transgender woman: 1 (20%)
24	Nagarakanti et al.^45^	USA	277	23	Median (IQR): 59 years (51–67)	Male: 14 (60.8), female: 9(39.2)
25	Okoh et al.^46^	USA	27	27	Median (IQR): 58 years (50–67)	Male: 15 (55.5%), female: 12 (44.5%)
26	Patel et al.^47^	USA	1	1	58 years	Male: 1 (100%)
27	Ridgway et al.^64^	USA	5537	8	Not mentioned	Not mentioned
28	Ruan et al.^49^	China	4	4	38, 25, 46, 54 years	Male: 4 (100%)
29	Sachdev et al.^50^	USA	276,807	193	48 years (20–76)	Male: 176 (91.2%), female: 12 (6.2%) Transgender female: 5 (2.6%)
30	Stoeckle et al.^51^	USA	120	30	60.5 years (56.6–70.0)	Male: 24 (80%), female: 6 (20%)
31	Swaminathan et al.^52^	USA	6	6	Mean: 64 years (62, 59, 45, 74, 57, 87 years)	Male: 5 (83%), female: 1 (17%)
32	Tesoriero et al.^53^	USA	108,062	2988	<40 years: 492 pt 40–60 years: 1400 pt >60 years: 1096	Male: 2109 (70%), female: 879 (30%)
33	Toombs et al.^54^	UK	3	3	62, 46, 57 years	Male: 2 (66%), female: 1 (34%)
34	Vizcarra et al.^55^	Spain	1339	51	Mean: 53.3 years	Male: 43 (84%), Woman: 8 (16%)
35	Wang et al.^56^	China	1	1	37 years	Male: 1 (100%)
36	Wu et al.^57^	China	2	2	60 years	Male: 2 (100%)
37	Yang et al.^58^	China	56	3	31, 60, 29 years	Male: 3 (100%)
38	Zhang et al.^59^	China	2	2	24, 37 years	Male: 2 (100%)

**Table 3:** Clinical information of patients with COVID‐19‐HIV co‐infection in the reviewed studies

ID	Signs and symptoms	Duration of disease	Last CD4 count (cells/µl)	Last viral load	Last antiretroviral regimen	Comorbidities	Clinical outcome	Diagnostic parameters during COVID‐19	inflammatory markers	COVID‐19 Therapy
1	N/A	N/A	N/A	N/A	N/A	Hypertension 5290 (19. 3%), Chronic respiratory disease 1095 (4.0%), Chronic heart disease 1444 (5. 3%), Chronic liver disease 921 (3. 4%), Stroke or dementia 559 (2.0%), Other neurological disease 239 (0.9%), Organ transplant 72(0. 3%)	25 Death in HIV/COVID‐19 population	N/A	N/A	N/A
2	1. Fever/cough/malaise/headache 2. Fever/cough 3. Fever/cough/malaise/headache/dyspnea 4. Fever/cough/malaise/headache/dyspnea 5. Fever/cough/dyspnea	1. 13 years 2. 17 years 3. 7 years 4. 17 years 5. 3 months	1. 616 2. 445 3. 604 4. 1140 5. 13	N/A	1. ART at admission remained(TenofovirAlafenamide, Emtricitabine, Darunavir‐boosted Cobicistat) 2. Tenofovirdisoproxilfumarate, Emtricitabine plus Lopinavir‐boosted Litonavir (ongoing) 3. Tenofovirdisoproxilfumarate, Emtricitabine plus Lopinavir‐boosted Ritonavir (for 3 days) 4. Tenofovirdisoproxilfumarate, Emtricitabine plus Lopinavir‐boosted Ritonavir (for 14 days) 5. TenofovirAlafenamide, Emtricitabine, Darunavir‐boosted Cobicistat(ongoing)	1. None 2. Hypothyroidism 3. None 4. Asthma 5. None	1. Cured 2. Still at the hospital 3. Cured 4. Cured 5. Cured	N/A	1. CRP, Ferritin: not done 2. CRP: 30 mg/dl, ferritin:1020 ng/ml 3. CRP:0.72 mg/dl, ferritin: not done 4. CRP: 0.43 mg/dl, ferritin: 1044 ng/ml 5. CRP:40 mg/dl, ferritin: 866(ng/ml)	Interferon beta‐1b hydroxychloroquine Meropenem linezolid Tocilizumab Azithromycin Azithromycin Cefixime Inhaled corticosteroids hydroxychloroquine Azithromycin ceftaroline fosamil co‐trimoxazole corticosteroids
3	Fever: 66.1% Cough: 66.1% Dyspnea: 46.8% Anosmia: 11.3% Ageusia: 9.7% Diarrhea: 22.6% Headache: 14.5% Weakness: 25.8% Myalgia/arthralgia: 24.2%	HIV infection time (years): 10.8 (median) (6.5–16.5)	N/A	< 50 copies/ml	PI‐based therapy: 9.8% INSTI‐based therapy: 63.9% NNRTIs‐based therapy: 26.2% TDF‐containing regimen: 14.8% TFV (TAF or TDF)‐containing regimen:26. 2%	Hypertension:19% DM:9. 5% Overweight :13. 1% Cardiovascular disease:12. 7% COPD:4. 8% chronic Kidney disease (crcl<30 ml/min):3. 2% Smoker:48. 2%	Global mortality rate:3. 22% Hospitalization rate:48. 4%	CD4(cell/mm^3^): 605 (Median) 391–921 Nadir CD4 < 200 (cell/mm^3^): 26.7%	Ferritin>1000(mcg/L):25%	Hydroxychloroquine: 49. 2% Corticosteroids: 19% LMWH: 46% Cyclosporine: 12,7% Tocilizumab: 6. 3%
4	Cough: 7 (77.8%) Myalgia: 7 (77.8%) Fatigue: 9 (100%) Anosmia and/or ageusia: 3 (33.3%) Dyspnea: 2 (22.2%)	Median (IQR): 21.4 years (13.6–29.2) N/A	Median (IQR): 258 (156–343)(cells/mm^3^)	Plasma HIV RNA ranged between 66 and 1240 copies/ml, and 7 patients had HIV RNA < 200 copies/ml (77.8%)	1 boosted protease inhibitor (PI) in 3 cases, 1 integrase strand transfer inhibitor in 4, and 1 nonnucleoside reverse transcriptase inhibitor in 2	Arterial hypertension, 6 (66. 7%) Diabetes mellitus, 2 (22. 2%) BMI > 30 Kg/m2, 1(11. 1%) COPD, 1 (11. 1%)	Recovery: 9(100%)	Six subjects had CD4 + lymphocyte count ranging between 200 and 350 cells/mm^3^, and 3 subjects had CD4 + lymphocyte count <200 cells/mm^3^	N/A	N/A
5	Fever > 38°C, cough, fatigue, myalgia, and tachypnea	N/A	Above 350 cells/mm^3^: 22 individuals (85%)	Undetectable HIV viral load in all patients	6 patients (23%): PI‐based cART (darunavir–Cobicistat in 5 cases and darunavir–ritonavir in one case). Sixteen patients: (61.5%): cART (tenofovirdisoproxilfumarate or tenofovirAlafenamide)	N/A	Recovery: 26(100%)	N/A	CRP (mg/dL), median:4. 2 (0. 71–9. 2)	Hydroxychloroquine: 50% Enoxaparin: 23%
6	Fever, myalgia, nausea, abdominal pain, diarrhea, hyposmia, hypogeusia, cough, dyspnea	15 years	CD4:426 VL: Undetectable	N/A	Tenofovir (TDF), lamivudine (3TC), and dolutegravir (DTG)	Hypertension	Discharged in good conditions	N/A	CRP:65. 5 LDH:316	HCQ + Azithromycin
7	Fever, fatigue	23 years	528 cells/π	N/A	Darunavir/cobicist	N/A	Cured	HIV‐1 viral load:below level of detection (<37 HIV‐1 RNA copies/ml) CD4 count: 242cells/ml, CD8 count: 336cells/ml	IL‐6: 50. 98 pg/ml	N/A
8	Headache (23%), Myalgias (19%), Shortness of breath (14%) Chills (12%), Fever (6%) and Loss of taste or smell (6%)	N/A	682 cells/mm^3^ (median)	Undetectable viral load: 74%	N/A	N/A	Recovered and symptom free: 71. 1% Feeling better but not completely recovered:26. 3% Not feeling better:2. 6%	Median CD4 cell count:682 cells/mm^3^; 74% had undetectable HIV viral loads load (<20 copies/mL).	N/A	N/A
9	N/A	N/A	N/A	N/A	Abacavir or Zidovudin Tenofovir Efavirenz Lopinavir Atazanavir Dolutegravir	Diabetes Hypertension Chronic kidney disease Chronic pulmonary disease/asthma Tuberculosis	Died:2. 8%	N/A	N/A	N/A
10	N/A	N/A	N/A	N/A	Tenofovirdisoproxilfumarate/emtricitabine: 12 395 (16%) TenofovirAlafenamide/emtricitabine: 25 570 (33%) Abacavir/lamivudine 20 105 (26%) Other regimens 19,520 (25%) Third drug: NNRTI 15 733 (21%) Protease inhibitor 14,267 (19%) Integrase inhibitor 37,622 (50%) Other 9968 (10%)	_	Age and gender standardized mortality from COVID‐19 in HIV‐positive persons (3. 7 per 10 000)	N/A	N/A	N/A
11	N/A	N/A	Median (IQR): 583 (474– 773)	HIV viral load < 40 copies: 96.2%	Protease inhibitors based; 9 (16.7%) Darunavir: 6 (11.1%) Atazanavir: 2 (3.7%) Lopinavir:1 (1.9%) Non‐nucleoside inhibitors based: 25 (46.3%) Nucleoside inhibitors based: 43 (79.6%) Integrase inhibitors: 33 (61.1%)	Diabetes: 5 (9. 3%) hypertension:16 (29. 6%) Other cardiac disease: 4 (7. 4%) Renal insufficiency (crcl<60 ml/min):3 (5. 6%) Respiratory disease (asthma, chronic bronchitis):5 (9. 3%) Cirrhosis: 1 (1. 9%) Active cancer:2 (3. 7%) Cardiovascular comorbidities:25 (46. 3%)	Cured: 43 (86%) Still hospitalized: 1(2%) Not cured: 5(10%) death:1(2%)	RNA HIV < 40 copies/ml: 51 (96. 2%)	N/A	N/A
12	HIV + group: Fever: (82.5%) Myalgia: (26.9%) Headache: (18.8%) Cough: (79.3%) Dyspnea: (72.7%) Chest pain: (22.9%) Sore throat: (14%) Wheeze: (5.9%) Rhinorrhea: (3.1%) Diarrhea: (25.9%) Nausea or vomiting: (21.9%) Abdominal pain: (12.5%) Fatigue: (43.9%)	N/A	N/A	N/A	N/A	HIV + group: Chronic pulmonary diseasea: (10. 8%) Asthma: (10. 3%) Chronic kidney disease: (18. 1%) Diabetes, no complications: (13. 7%) Diabetes, with complications: (7. 7%) Obesity: (17%) Chronic neurological disorder: (6. 9%) Dementia: (2. 5%) Mild liver disease: (2. 5%) Moderate/severe liver disease: (5. 1%) Rheumatological disease: (5. 1%) Malnutrition:(4. 5%)	N/A	N/A	CRP: (median) 107 mg/L	N/A
13	Fever: 7 (50%) Shortness of breath: 7 (50%) Cough:10 (70%) Diarrhea: 4 (29%) Anosmia, ageusia: 4 (29%)	N/A	Median (IQR): 519.5 (21–1756)	11 patients <20 1patient: 25 1patient: 36 1patient: 1646 (copies/ml)	N/A N/A	Obesity (N = 8; 57%) hypertension (N = 8; 57%) diabetes (N = 6; 43%), chronic kidney disease (N = 5; 36%) and ESRD requiring hemodialysis (N = 2; 14%) CHF COPD	5 patients died	N/A N/A	CRP: 6patients; not available Median:11. 65(2.1–21.5 mg/dl)	N/A
14	N/A	Average:2740 ± 1140 days	CD4 count > 200/µl: 9 (pts with COVID19‐HIV coinfection)	9 pts had undetectable viral loads	(1406, 82.7%): (NRTIs) and (NNRTIs) 172 (10.1%): LPV/r‐based ART, 87(5.1%): integrase inhibitors (INI)‐based ART (62 dolutegravir‐based, 19 elvitegravir/cobicista‐based, 4 raltegravir‐based, 2 bictegravir‐based). 36 individuals (2.1%) were still treatment naïve	N/A	N/A	Nine out of the 11 COVID‐19/AIDS patients had relatively high CD4 count (>200/μl) and undetectable HIV viral load (<20 copies/ml).	N/A	N/A
15	Cough: 25 (78%) Fever: 22 (69%) Arthralgia/myalgia: 7 (22%) Headache: 7 (22%) Sore throat: 7 (22%) Sinusitis and anosmia: 6 (19%) for each	N/A	Median (IQR): 670/mm^3^ (69–1715/mm^3^)	In 30/32 cases: below 50 copies/ml	Nucleoside reverse transcriptase inhibitors (NRTIs) in 31 patients, integrase strand transfer inhibitors (INSTI) in 20, protease inhibitors (PI) in 4 and non‐NRTIs in 9 cases. NRTIs were mainly tenofoviralafenamide (16 cases), tenofovirdisoproxilfumarate (6 cases) and a cytidine analog, either emtricitabine (*n* = 22) or lamivudine (*n* = 9)	Documented in 20/33 (60%) patients: arterial hypertension(n = 10) COPD:(n = 6) diabetes mellitus(n = 4) cardiovascular disease (n = 3) renal insufficiency (n = 2) Coinfection with hepatitis B (n = 5) resolved hepatitis B (hepatitis B surface antigen negative): (n = 5) chronic hepatitis B(n = 1). cured hepatitis C:(n = 1)	Recovered: 29/32 of patients (91%) Died:3	N/A	N/A	N/A
16	Fever: 61 (65.6%) cough: 71 (76.3%) shortness of breath: 57 (61.3%) Altered mental status: 10/93 (10.8%) Congestion: 13 (14%) Sore throat: 18 (19.4%) Myalgia: 33 (35.5%) Anosmia: 2 (2.2%) Diarrhea: 18/93 (19.4%) Headache: 17 (18.3%)	Median: 20 years (15–26, *n* = 57)	Median (IQR): 554 (339–752) (*n* = 64)	Plasma HIV RNA < 50 copies/ml (*n* = 68): 57 (83.8%)	62/89 (69.6%) were on an antiretroviral therapy (ART) regimen: tenofovir 12/89 (13.5%) were on a regimen including a protease inhibitor	Autoimmune disease: 4(4. 3%) Cancer: 8(8. 6%) Diabetes: 32(34. 4%) Heart disease, CAD, or CHF: 17(18. 3%) Hypertension:49(52. 7%) Lung disease, asthma, or COPD:25(26. 9%) Chronic kidney disease:16(17. 2%) End‐stage renal disease:7(7. 5%) Solid organ transplant:5 (5. 4%)	Recovered:53 Died:19	CD4 + : 220 cells/uL (n = 53) (132–372) Plasma HIV RNA < 50 copies/mL: (n = 46) 41(89. 1%)	CRP (n = 69):Median CRP,137 mg/L (91. 7–240) Median interleukin‐6:57. 6 pg/mL (30. 5‐130. 9)(n = 48) Interleukin‐6 elevated: 47(97. 9%) Interleukin‐8 (n = 22) Median interleukin‐8:42. 2 pg/mL (30. 6‐68. 5) Interleukin‐8 elevated: 22/22 (100%) TNF‐alpha (n = 22) Median TNF‐alpha, 21. 8 pg/mL (16.3–38.5) TNF‐alpha elevated: 11/22 (50%) IL‐1‐beta (n = 21) Median IL‐1‐beta:0. 3 pg/mL (0.3–0. 5) IL‐1‐beta elevated: 0(0)	N/A
17	N/A	N/A	298/ml (135–542), *N* = 19	Only 1 patient had a viral load>50 copies/ml	N/A	N/A	Mortality rate:28. 6% In HIV group	N/A	HIV group: Ferritin:679 ng/mL (338–1446) N = 19 CRP:154. 48 ± 94. 44 mg/L,N = 18 Lactate dehydrogenase:449. 4 ± 239. 8 U/L, N = 5	N/A
18	Cough, sputum Chilling, myalgia Rhinorrhea, sore throat, loss of taste and smell	5 years	N/A	N/A	Genvoya®: elvitegravir/cobicistat/emtricitabine/tenofovir	N/A	Improved and discharged (with persistent positive PCR)	CD 4 count: 555/mm^3^ CD8count: 1387/mm^3^ HIV RNA: < 20 copies/mL	ESR: 25 mm/hr(elevated)	N/A
19	N/A	Median: 5 years (1–14 years)	557 cells/mm^3^	HIV viral load log: median:4.93 copies/ml (4.2–6)	82.3% on ART regimen	52. 9%(18 pts)had comorbidities: Cardiovascular disease:5 Chronic lung disease:2 Diabetes:2 Hypertension:2 Other:7	Fully recovered:26 Died:2 Still in hospital:6	Undetectable HIV RNA:in 54. 5%	N/A	N/A
20	Fevers, chills, nasal congestion, and mild cough	23 years	435 cells/µl	< 20 copies/ml	Dolutegravir, emtricitabine, and tenofovir alafenamide.	hypertension, asthma, steatohepatitis, and resolved hepatitis B infection. HIV‐associated nephropathy)/focal segmental glomerulosclerosis (FSGS)	Cured	CD4: 395 cells/µl HIV RNA: < 20 copies/mL	N/A	No specific treatment
21	Fatigue, fever, chills, and pharyngeal pain	8	Nadir CD4 + count: 224	Remained undetectable from 2013	Efavirenz 600 mg, zidovudine 300 mg, and lamivudine 150 mg	N/A	Cured	N/A	N/A	Interferon atomization (5 million bid) Ribavirin (150 mg TID) Abidol (200 mg TID)
22	N/A	N/A	N/A	N/A	N/A	Hypertension: 74 Diabetes: 46 Dyslipidemia: 55 Heart Failure: 15 CKD: 39	Admission: 36 Intubated: 19 Death: 23	N/A	N/A	N/A
23	Asymptomatic: 2 Fever: 3 Dry cough: 3 Shortness of breath: 1 Myalgia: 1	Pt1: NA Pt2: 22 years Pt3: 3 years Pt4: 1 years Pt5: 20 years	1:438 2:112 3:219 4:127 5:352 CD4 in hosiptal	<30: 5 All patient less than 30	DTG + DRV/r: 1 DTG + DRV/c: 1 TDF/FTC + EFV: 1 TDF/FTC + DTG: 1 TAF/FTC/RPV: 1 (5 patients)	Asthma: 1 Cardiomyopathy:1 HBV:1 No Comorbidity:2	Cure: 5	1:438 2:1127 3:219 4:127 5:352 CD4 in admission	1: ferritin=241; LDH = 207; Lymphocyte=1252 2: ferritin=NA; LDH = 227; Lymph=2514 3: Ferrtitin= NA; LDH = 128; Lymph=2306 4: ferritin= 256; LDH = 154; Lymph=644 5: Ferritin=254; LDH = 167; Lymph=1443	Pt1: HCQ + Tocilizumab + Methylprednisolone Pt2,3,4: HCQ Pt5: No specific treatment
24	Cough: 20 Fever: 18 Dyspnea: 17 Myalgia: 11 Diarrhea: 4	N/A	N/A	N/A	Integrase based: 8 NNRTI: 5 PI + integrase based: 6 Not available: 2 Protease inhibitor based: 1 NNRTI + integrase based: 1	Hypertension:15 Diabetes mellitus:7 Chronic kidney disease:11 Dialysis:5 Coronary artery disease: 2 COPD:1	Mortality:3 cured:21	CD4 > 200: 16/19 pt 3/19 CD4 = 10 1 pt CD4 = 116 1 pt CD4 = 179 1 pt Undetectable Viral Load: 16/18 pt HIV VL = 26900 1 pt HIV VL > 2million 1 pt	Lymphocyte:1056 (16%) Procalcitonin (ng/ml) 0. 25 (0.07, 0. 39) LDH = 557	Hydroxychloroquine: 11 Azithromycine:9 Cefriaxone: 8 Remdesivir: 0 Steroids: 5 Tocolizumab:5
25	Cough: 18 Fever: 17, Dyspnea: 17, Fatigue: 13, Myalgias: 9, Diarrhea: 4, Nausea/vomiting: 4	N/A	Median (IQR): 551 (286–710)	<20: 11 20–120:15 >120:1	Integrase based: 9 NNRTI: 5 PI + integrase: 5 Not available: 4 NNRTI + integrase: 3 PI based 1	Hypertension: 16 Diabetes mellitus:9 CKD:10 Dialysis 6 CHF: 3 CAD: 1 COPD: 0	ICU Care: 3 pt Death: 2 pt	N/A	Lymph= 17% Procalcitonin, m/L 0. 26 (0. 08–0. 41)	Hydroxychloroquine: 7 Azithromycine: 8 Remdesivir: 0 Steroids: 1 Tocilizumab: 0
26	Weakness, anorexia, and diarrhea for 2 weeks	N/A	CD4 = 497 (43%)	N/A	Emtricitabine (200 mg) and tenofovir (25 mg) every 24 h, atazanavir (300 mg) every 24 h, and ritonavir (100 mg) every 24 h	chronic bronchitis hypertension	Recovered(Discharged)	N/A	Lymph: 23% = 1334	Hydroxychloroquine + Azithromycin + Zinc Sulfate
27	N/A	N/A	N/A	N/A	N/A	N/A	Hospitalized; 6/8 pt ICU admission: 1/8 pt Death: 0/8 pt	N/A	N/A	N/A
28	Pt1: cough, fever, dyspnea Pt2: fever, cough, and dyspnea Pt3: fever, cough. Pt4: fever, cough, and dyspnea	Pt1: 1 year Pt2: 1 year Pt3: 5 years Pt4: 4 years	Pt1: CD4 = 34 Pt2: CD4 = 12 Pt3: CD4 = 540 Pt4: CD4 = 743	N/A	1: No therapy 2: No therapy 3: EFV/3TC/TDF 4: EFV/3TC/TDF	Pt1,2,3 = no comorbidity Pt4: Hypertension, Diabetes, CHD	1,2= severe 3,4=moderate All Discharged	_	Pt1:Procalcitonin = 0.05 Pt2:ESR = NA Procalcitonin=0.05 Pt3:ESR = 3 Procalcitonin=0.05 Pt4:ESR = 54 Procalcitonin=0.06	N/A
29	Cough (38.8%), fever (33.9%), rhinorrhea (25.7%), myalgias (28.4%), headache (26.8%), chills (21.9%), shortness of breath (15.3%), sore throat (15.3%) loss of taste/smell (19.1%)	Year of HIV diagnosis 1985–2010 133 (68. 9%) 2011–2015 26 (13. 5%) 2016–2020 34 (17. 6%)	Last CD4 count result: 121 (62.7%) CD4 count > 500 cells/mm 60 (31.1%) CD4 count of 200–500, 12 (6.2%) CD4 count <200	N/A	Increased incidence of SARS‐CoV‐2 infection among HIV compared with people without HIV in San Francisco from the date community transmission was reported	Patients Interviewed (n = 183) Any comorbidity:78 (42. 6%) Lung disease: 8 (4. 4%) Diabetes: 9 (4. 9%) Cardiovascular disease; 13 (7. 1%) Chronic renal disease 2 (1. 1%) Liver disease: 3 (1. 6%) Other comorbidities No 144 (78. 7%) Yes 39 (21. 3%)	Outcomes of Patients Interviewed (n = 183) Hospitalized: 14 (7. 6%) ICU admission: 2 (1. 1%) Deceased: 0	N/A	N/A	N/A
30	Fever 17 (57%) Cough 21 (70%), Sputum production 1 (3%) Dyspnea 20 (67%) Sore throat 0 (0%) Rhinorrhea or nasal congestion 1 (3%) Headache 3 (10%) Myalgias 4 (13%) Nausea or vomiting 5 (17%) Diarrhea 10 (33%) Abdominal pain 3 (10%) Chest pain 3 (10%) Anosmia 1 (3%) Ageusia 2 (7%)	N/A	CD4 count, median (IQR): 332 (123–526) 7 patients CD4 < 200 count CD4:CD8 ratio, median (IQR) 0.7 (0.3–1.0) HIV viral load, median (IQR): 0(0) All patients had undetectable viral loads	N/A	N/A	Hypertension 12 (40%) DM: 8 (27%) Coronary artery disease 2 (7%) Stroke 0 (0%) Chronic kidney disease 0 (0%) End‐stage renal disease 2 (7%) Chronic obstructive pulmonary disease 4 (13%) Asthma 3 (10%) Cirrhosis 1 (3%) Chronic hepatitis B 6 (20%)	Hypoxemic: 15/30 pt Intubation: 4/30 pt ICU admission: 4/40 pt Discharged: 24/30 pt Death: 2/30 pt		Absolute lymphocyte count, median 900 CRP: 7.6 (2. 8–16. 5) Procalcitonin, median (IQR), ng/mL 0. 16 (0.06–0. 3) d‐dimer, median (IQR), ng/mL 1021 (427–3145) Lactate dehydrogenase, median (IQR), U/L 410 (283. 5–535)	Hydroxychloroquine: (67%) systemic corticosteroid s: (13%) Remdesivir:(0)
31	N/A	N/A	Last CD4 (cells/mm^3^): 491,1500, 500,772,678, 651 viral load (copies/ml) 10,000 (1 pt) Undetectable (4 pt) CD4: mean 765		RPV/RAL/3TC ABC/EFV/3TC BIC/TAF/FTC BIC/TAF/FTC EVG‐c/TAF/FTC EFV/TDF/FTC	Hypertension: 4 pt Coronary Disease:2 pt Diabetes: 3Pt ESRD:1 pt COPD:2 pt {Total: 6 pt)	2 Patients: Expired Discharged:4 pt »> Mild:1 pt Moderate:2 pt Severe: 1 pt		Lymphocyte: 1010,1770,1220,780,560,620 LDH: 338,520,214 508,499,258 CRP (mg/L): 274,243. 8, NA 178. 6,74,277. 2 Procalcitonin: (ng/ml) 5. 83,2. 74, NA, NA,0. 1,0. 42	Pt1: Hydroxychloroquine Pt2: Hydroxychloroquine + Steroid Pt3,4: No specific treatment Pt5:Hydroxychloroqune Pt6: Hydroxychloroquine
32	N/A	N/A	Stage 1 HIV: 1774 Stage 2 HIV: 843 Stage 3 HIV: 270 Others: 101 Viral suppression: Yes: 2628 No: 267 Stage 1: CD4 ≥ 500 cells/mm^3^ or ≥26% of total lymphocytes (age ≥ 6 years), CD4 ≥ 1000 cells/mm^3^ or ≥30% of total lymphocytes (age 1–5 years); Stage 2: CD4 200–499 cells/mm^3^ or 14%–25% of total lymphocytes (age≥6 years), CD4 500–999 cells/mm^3^ or 22%–29% of total lymphocytes (age 1–5 years); Stage 3: CD4	N/A	N/A	N/A	Total Case: 2988 Hospitalized: 896 Death: 207	N/A	N/A	N/A
33	N/A	Pt1:18 years Pt2:7 years Pt3:8 years	CD4: Pt1:180 Pt2:50 Pt3:890 VL: Pt1,3: Not detected Pt2: >1 million	N/A	Pt1: Raltegravir 400 mg BD Lamivudine 50 mg OD Abacavir 600 mg OD Pt2: Truvada200/245 OD Dolutegravir 50 mg OD Pt3: Descovy 200 mg/25 mg Nevirapine 400 mg OD	Pt1: Hypertension, ESRD, Renal transplant, Diabetes Pt2: G6PD Pt3:Hypertension, Diabetes, Obesity	Pt1: Death Pt2,3: CPAP (Discharged later)	N/A	Lymphocyte: Pt1: 230 Pt2: 1130 Pt3: 1100 CRP, mg/dL Pt1:260 Pt2:51 Pt3:78	Pt1,2: Prednisolone Pt3: No Specific Treatment
34	Fever: 36 (71%) Sore throat: 10 (20%) Cough: 37 (73%) Dyspnea: 28 (55%) Anosmia or ageusia: 7 (14%) Myalgia: 17 (33%) Headache: 13 (25%) Fatigue: 27 (53%) Diarrhea: 11 (22%)	19.5 years (9.3–28.6)	Recent CD4: 565 (296–782) CD4 < 200: 6 pt (12%) Last HIV‐RNA < 50:50(98%)	N/A	Protease inhibitors 11 (22%) NNRTI 8 (16%) INSTI 41 (80%) Tenofovir (TAF or TDF) 37 (73%)	Any 32 (63%) Hypertension 18 (35%) Cardiovascular disease 14 (27%) Diabetes 7 (14%) Chronic kidney disease 6 (12%) Chronic liver disease 24 (47%) Chronic respiratory disease 13 (25%)	Total Case: 51 Admit: 28 (55%)=> 22 noncritical disease 6 critical disease 2 deaths 21 recovered 5 still admitted	N/A	Lymphocyte: 1200 (800‐1800) Serum ferritin, ng/mL (n = 7) 972 (366–2791) Procalcitonin, ng/mL: 0.08 (0.04–0.13) C‐reactive protein, mg/L 32.1 (7.1–120.6)	12 (24%) of 51: no specific antiviral therapy hydroxychloroquine^23^: azithromycin^18^ ritonavir‐boosted lopinavir (14 boosted darunavir: 8 1: Remdesivir 15 (38%): systemic corticosteroids at doses of 1.0–1.5 mg/kg per day of prednisone or equivalent, 4 (10%): tocilizumab
35	Fever, dry cough, chest pain	N/A	CD4 cell count = 34/ul, CD8 cell count = 737/ul CD4/CD8 = 0.05	N/A	N/A	N/A	1 Pt Mechanical Ventilaton + ICU admission (Still admitted)	N/A	Lymphocyte: 1550 CRP:96. 5 LDH:423	Arabidol then Methyprednisolone then Tocilizumab
36	Pt1: Myalgia, fever, dyspnea, productive cough Pt2: Fever, nonproductive cough, myalgia, sore throat, shortness of breath, diarrhea	Pt1: 6 years Pt2: New diagnosed	N/A	N/A	Pt1: Tenofovirdisoproxilfumarate, lamivudine and efavirenz Pt2 ‐	Pt1: stage IV diffuse large B‐cell lymphoma, pulmonary tuberculosis, Diabetes Pt2 ‐	2 Pt: Moderately ill and discharged	N/A	Pt1: Lymph= 900 Procalcitonin (PCT) = 0. 19 ng/ml; C‐reactive protein (CRP) = 191. 21 mg/L Pt2: Lymph= 670 Procalcitonin= 0.05	Pt1: Oseltamivir Pt2: Ribavirin + Umifenovir)
37	N/A	N/A	CD4: Pt1 = 420 Pt2 = 550 Pt3 = 21	N/A	Pt1: AZT/3TC/NVP Pt2: TDF/3TC/EFV Pt3: STRIBILD	N/A	N/A	N/A	Lymphocyte count: Pt1: 950 Pt2: 900 Pt3: 430	N/A
38	Pt1: Fever, fatigue, poor appetite, shortness of breath, sore throat Pt2: Fever, wheezing, palpitation, dyspnea, chest pain	Pt1,2= New HIV	Pt1: CD4 = 13 Pt2: CD4 = 23	Pt1 – Pt2 –	N/A	N/A	Pt1: the patient was transferredto a designated hospital for further treatment (moderate to severe disease) > Discharged Pt2: Severe disease (Transferred to another hospital) > Discharged	N/A	Pt1: Lymph=1080 CRP = 39. 71 Procalcitonin: 0.03 Pt2: Lymphocyte=1550 CRP = 96. 51	Pt1: Arbidol then Tocilizumab Pt2: Arbidol the Methylprednisolone then Tocilizumab

Among the total studied population of 3,993,400 COVID‐19 patients, 89,343 patients had COVID19‐HIV coinfection, among which 72% (ranged from 42.3% to 100%) were male, 0.01% (11 patients) were transgender. Patients' gender was not available in two studies. Patients' age was above 20 years in all studies. As the review revealed, nine studies did not mention the ART regimen for HIV + patients. At the time of the COVID‐19 diagnosis, the most common symptoms were fever, cough, myalgia, and headache. Additionally, most of the patients had various comorbidities such as hypertension, diabetes mellitus, asthma, renal insufficiency, cardiovascular disease, etc.

Laboratory values including CRP, ferritin, and Interleukins levels were available in about two‐third of the studies. But in almost all the studies with inflammatory tests results, the values were elevated. In most of the studies, all HIV patients completely recovered from the COVID‐19 infection. In 8 studies, mortality was reported ranging from 1% to 36%. Although CD4 count was not recorded in all the studies, the minimum level was reported as 12 cells/µl.

## 4 DISCUSSION

We found that HIV patients at advanced stages (3 or 4) of the disease with low CD4 count and weak immune systems show less severe COVID‐19 symptoms. However, some studies showed controversial results which contradict our primary hypothesis. The main reason for these contradictory results was the scarcity of existent literature and inconsistency of evidence that limited our ability to address and reasonably argue our main hypothesis. This may also be due to simultaneous symptoms and underlying comorbidities that come along at advanced stages of HIV infections and could perplex and obscure the typical presentation of COVID‐19 in such patients. Despite these contradictory results, the majority of studies included in this review indicate mild or no typical symptoms of COVID‐19 in HIV patients, particularly in those at the advanced stages of HIV disease. This review also found an unexpected high recovery rate in these patients after COVID‐19 infection, which contradicts the common knowledge of higher morbidity and mortality rate in immunocompromised patients.

SARS‐CoV‐2 is a new strain of coronavirus, which is the causal agent of COVID‐19. The usual symptoms of COVID‐19 are fever, cough, headache, shortness of breath, tiredness, loss of taste or smell, and gastrointestinal symptoms such as diarrhea, anorexia, nausea, and abdominal pain,^1^ of which, many are due to the cytokine storm caused by the host's immune system. To control these symptoms, corticosteroids have been used now and then by clinicians around the world which indicates the substantial role of immune system function interfered with SARS‐CoV‐2. Application of Canakinumab, a humanized monoclonal antibody against IL‐1β in a sub‐group of hospitalized patients with COVID‐19 and subsequent swift reduction in the systemic inflammatory response and oxygenation improvement by Claudio Ucciferri et al. also manifests the fundamental role of the immune system and inflammatory cytokines in the SARS‐CoV‐2 pathophysiology.^65^, ^66^ The human immunodeficiency virus targets the body's natural immune system and causes immune deficiency. This immune deficiency can lower the severity of the immune system reactions such as cytokine storms and subsequently the accompanying symptoms. This could explain the milder symptoms, lower morbidity, and less mortality among HIV‐positive patients infected by COVID‐19 as the primary fatal condition in COVID patients is caused by the cytokine storm which subsequently leads to multiorgan dysfunction and death. The hypothesis was supported by some of the included studies in the present review, while, some contradictory results were also observed.

In the present review, a CD4 count less than 500 was assumed as a cutoff point. Thus, the patients with a mean CD4 count less than 500 were considered immune‐deficient and assumed to be at advanced stage patients. In the study conducted by Calza et al. all the nine HIV‐positive patients with CD4 count less than 258 fully recovered, which supports the hypothesis of our study.^26^ Additionally, in Kumar et al.'s and Patel RH.'s study, the patients had a CD4 count less than 500 and again completely recovered.^41^, ^47^ Similar findings were reported by Mondi A et al.^44^ In contrast, in Karmen‐Tuohy et al.' study contradictory results were reported. Although the median CD4 count of patients was lower than 500 and the patients were immunodeficient, the mortality rate was higher (28%).^38^ Likewise, in the study by Blanco et al., all the patients with CD4 count > 500 were cured, but one of the two patients with CD4 count < 500 remained in the hospital due to the severity of illness that may have been due to comorbidities as is reported by authors.^23^ However, the findings of the Ruan L et al' study were completely against our hypothesis. In this study, all the patients with CD4 count > 500 experienced moderate‐severity clinical outcomes; while, all the patients with CD4 count < 500 had severe clinical outcomes.^49^

### 4.1 Limitation

Despite the limited available evidence, the findings of the present review authenticate the primary hypothesis arguing less severe clinical outcomes in HIV patients at the advanced stages. Although this could be mainly due to the inability of the immune system in HIV patients to provoke the cytokine storm, which is believed to be the main responsible event for severe clinical outcomes in COVID patients, the contradictory results inform future studies to explore further the possible underlying causes of such an observation.

## 5 CONCLUSION

In conclusion, the results of the present study suggest that HIV patients at advanced stages (3 or 4) of the disease, when CD4 counts are low and their immune system is compromised, manifest less severe symptoms and less mortality following COVID‐19. This has been attributed to the inability of HIV‐positive individuals' immune systems in provoking the cytokine storm that caused the severe clinical outcome in COVID patients. By a similar mechanism, it seems that corticosteroids mitigate the severity of symptoms in COVID patients with a healthy immune system.

## CONFLICT OF INTERESTS

The authors declare that there are no conflict of interests.

## AUTHOR CONTRIBUTIONS

*Conception and design of the study*: Esmaeil Mehraeen, SeyedAhmad SeyedAlinaghi; *acquisition of data*: Amirali Karimi, Seyed Peyman Mirghaderi, Amirata Fakhfouri; *analysis and interpretation of data*: Hengameh Mojdeganlou, Alireza Barzegary; *drafting the article*: Esmaeil Mehraeen, Mehrzad MohsseniPour, Solmaz Saeidi; *revising it critically for important intellectual content*: SeyedAhmad SeyedAlinaghi, Armin Razi; *final approval of the version to be submitted*: Esmaeil Mehraeen, Amir Masoud Afsahi, Omid Dadras, Marcarious M. Tantuoyir.

## ETHICS APPROVAL AND CONSENT TO PARTICIPATE

Not applicable. This research did not receive any specific grant from funding agencies in the public, commercial, or not‐for‐profit sectors.

## Data Availability

The authors stated that all information provided in this article could be shared.
